# Publication of observational studies making claims of causation over time

**DOI:** 10.1016/j.conctc.2024.101327

**Published:** 2024-06-17

**Authors:** Alyson Haslam, Vinay Prasad

**Affiliations:** a Department of Epidemiology and Biostatistics, University of California San Francisco, 550 16th St., San Francisco, CA, CA 94158, USA; b Department of Medicine, University of California San Francisco, 550 16th St., San Francisco, CA, CA 94158, USA

## Abstract

To examine methodology characteristics over time and investigate research impact before and after the start of the COVID-19 era, we analyzed original articles published in *The New England Journal of Medicine* between October 26, 2017 and August 27, 2022. April 1, 2020 was used as the defining date dividing before and after the COVID-19 era. Out of 1051 original articles, 515 (49 %) were before and 536 (51 %) were after the COVID-19 era. Two independent reviewers categorized and reconciled methodology into groups: “randomized trial” (715 articles), “uncontrolled experimental study” (128), “descriptive observational study” (168), and “observational study making a causal claim” (40). We extracted subsequent citations and Altmetric data for each article to assess impact.

The median number of social media shares was 2272 (IQR: 743–7821) for observational studies making a causal conclusion, compared to 306 (IQR: 70–606) for randomized trials (p-value=<0.001). The median Altmetric score for randomized COVID-19 trials (2421, IQR: 1063–3920) was not significantly different than that of COVID-19 observational studies making a causal claim (2583, IQR: 1513–6197, p-value = 0.42), but it was significantly lower than descriptive observational COVID-19 studies (4093, IQR: 2545–6823, p-value = 0.04).

We conclude that there has been a steady increase in the number and percentage of observational studies that make causal conclusions about the efficacy of an intervention. Research concerning COVID-19, regardless of methodology, has seen a sharp rise in dissemination as measured through Altmetric's social media score and subsequent citations.

## 1 Introduction

In addition to its impact on people, societies and populations, the COVID-19 pandemic altered scientific communication and dissemination. The pandemic necessitated the real time sharing of scientific results to broad audiences and changed many medical publishing norms.

Scientific output pertaining to COVID-19 grew quickly after the initial outbreak [1,2]. Preprint servers, a non-peer reviewed method to rapidly disseminate manuscripts, saw an influx of submissions. Over 700,000 scientists contributed to COVID-19 research, and there have been over 7000 COVID-19 papers on preprints [2,3]. At the same time, there were several high-profile retractions of COVID-19-related research [4,5].

Prior to the pandemic, observational research that sought to make causal claims was a topic of debate, though novel techniques – like the target trial framework– had been offered to transform real world evidence into reliable causal conclusions [[6], [7], [8], [9], [10], [11]]. Given the urgency of the pandemic, these methodologic approaches might have been more embraced by high impact journals that were previously critical. As such, we sought to examine the nature of studies – specifically the methodology – featured in the *New England Journal of Medicine (NEJM)* before and after the start of the pandemic.

## 2 Methods

We assessed the quality and nature of academic publications over time in a cross-sectional analysis. The NEJM was selected for review, as it has the highest impact journal in the clinical medical sciences.

### 2.1 Data collection

We reviewed all original research articles, including brief reports, published in the NEJM between October 26, 2017 and August 27, 2022. April 1, 2020 was used as the dividing point for defining pre- and post-COVID-19 timeframes. We extracted original articles from every weekly NEJM publication from 127 weeks before and after this time-point.

Variables extracted for analysis include date of publication, number of citations, trial identifiers, topic (COVID-19 versus non-COVID-19), number of authors, type of original research (brief report vs. full-length report). Trial identifiers were extracted and cross-referenced with ClinicalTrials.gov.

Type of each article was rated and classified into categories broadly based on the hierarchy of evidence framework for study design. We coded each article as being a “randomized trial”, “uncontrolled experimental study”, “descriptive observational study”, or “causal observational study”. Randomized studies were exclusively interventional studies with two or more randomized arms. Uncontrolled experimental studies included interventional trials that were single arm in design. We defined descriptive observational studies as non-interventional studies that reported descriptive observations about the world or a practice but did not make claims about efficacy. These studies could include reporting on safety. Observational efficacy studies making a causal claim included non-interventional observational studies that specifically claimed a causal conclusion or inference about the efficacy of a medical practice.

Two reviewers (A.H. & J.T.) independently categorized each of the remaining articles in accordance with the coding schema. Reviewers collaboratively reconciled discordant categories. If no consensus was reached a third reviewer (V.P.) assessed and provided final judgment. Articles measuring outcomes in non-human entities (primates, AI algorithms) were excluded.

Altmetric scores for each article were obtained through the Altmetric website, using the article's DOI number. Impact per article was measured through Altmetric data (Altmetric score, social media shares) and number of citations of the study. To account for time lag in publication, we divided the number of citations by weeks since publication to estimate the average weekly number of citations for each article.

COVID-19 topic was defined as having “COVID-19”, “SARS-CoV-2” or any COVID-19 vaccines directly named in the article title.

### 2.2 Analysis

R statistical software (version 4.2.2) was used for statistical analysis and data visualization. Kruskal-Wallis one-way ANOVA was used to detect a difference in the continuous independent variables (weeks since COVID-19, average weekly citations, Altmetric score, social media shares, number of authors) between study type categories. For analyzing differences in COVID-19-focused articles versus non-COVID-19 articles, a Wilcoxon rank sum test with continuity correction was used instead. Pearson's Chi-squared test was used for analyzing both methodology categories and COVID-19 articles against categorical independent variables (pre-versus post-COVID-19 era, COVID-19-focused articles, brief reports). In accordance with 45 CFR §46.102(f), this study was not submitted for institutional review board approval because it involved publicly available, non-patient data. Data were collected September 1, 2022–October 1, 2022.

## 3 Results

We identified 1053 original articles published in the NEJM between October 26, 2017 and August 27, 2022; 2 were excluded because of non-human subjects. Among 1051 NEJM articles that were analyzed, 515 (Table 1, 49 %) were published before the COVID-19 era and 536 (51 %) after COVID-19.

**Table 1:** Characteristics of original articles published in The New England Journal of Medicine between October 26, 2017 and August 27, 2022, overall and by study type.

	Overall, N = 1051	Randomized trial N = 715	Uncontrolled experimental N = 128	Descriptive observational, N = 168	Causal observational, N = 40	p-value^a^
Weeks since April 1, 2020, median (IQR)	2 (-62–65)	−3 (-62–60)	6 (-64–68)	−2 (-71–52)	90 (62–105)	<0.001
Time period, before/after COVID-19, n (%)						<0.001
Post	536 (51)	350 (49)	68 (53)	83 (49)	35 (88)	
Pre	515 (49)	365 (51)	60 (47)	85 (51)	5 (12)	
Covid-19 paper, n (%)	117 (11)	55 (7.7)	4 (3.1)	29 (17)	29 (72)	<0.001
Weekly citations, median (IQR)	1.20 (0.56–2.59)	1.22 (0.61–2.58)	1.21 (0.51–2.46)	0.77 (0.34–2.28)	1.92 (1.44–3.31)	<0.001
Altmetric score, median (IQR)	426 (240–818)	395 (242–725)	452 (233–797)	463 (172–1106)	1970 (931–5591)	<0.001
Social media shares, median (IQR)	317 (168–672)	306 (170–606)	256 (152–460)	321 (157–892)	2272 (743–7821)	<0.001
# of authors, n (%)	21 (13–30)	21 (14–30)	22 (16–32)	18 (11–31)	12 (9–26)	0.006
Brief report, n (%)	49 (4.7)	0 (0)	27 (21)	22 (13)	0 (0)	<0.001

^2^multi-choice allowed. May sum to more than 100 %.

a Kruskal-Wallis rank sum test; Pearson's Chi-squared test. p<0.05.

**Fig. 1:**
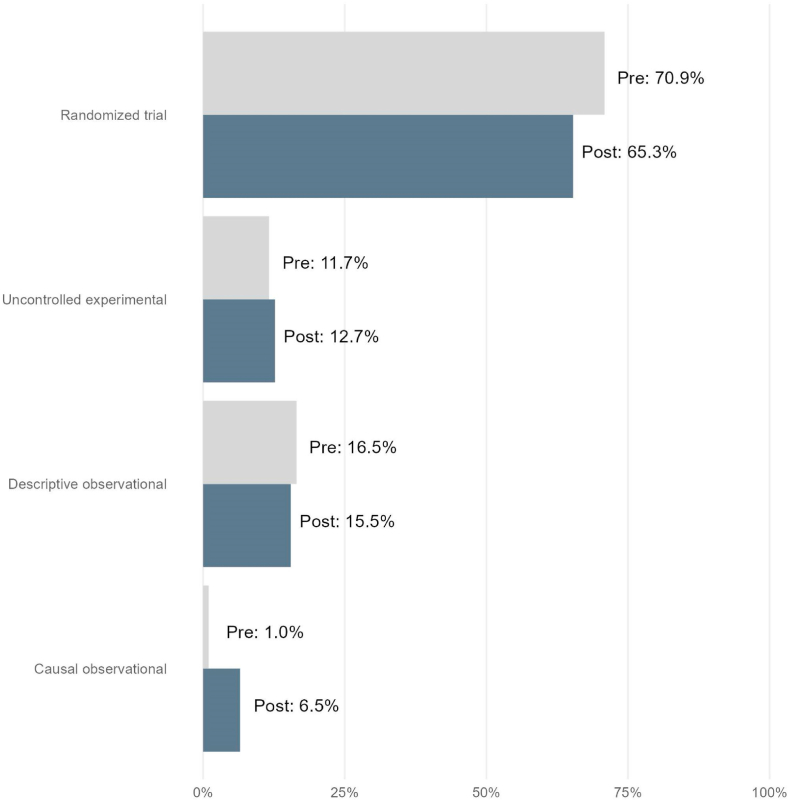
Percentage of original articles published in The New England Journal of Medicine, by study type and publication date (pre: October 26, 2017 through April 1, 2020 vs. post: April 1, 2020 through August 27, 2022).

Among the 515 articles published prior to the start of the COVID 19 pandemic, 70.9 % were randomized trials, 11.7 % uncontrolled experimental, 16.5 % were descriptive observational, and 1 % were observational studies making a causal claim. Among the 536 articles published after the start of the pandemic, 65.3 % were randomized trials, 12.7 % were uncontrolled experimental, 15.5 % descriptive observational, and 6.5 % observational studies making a causal claim. These results are presented graphically (Fig. 1). Differences in study type pre- and post-COVID-19 were significant (p-value <0.001).

**Fig. 2:**
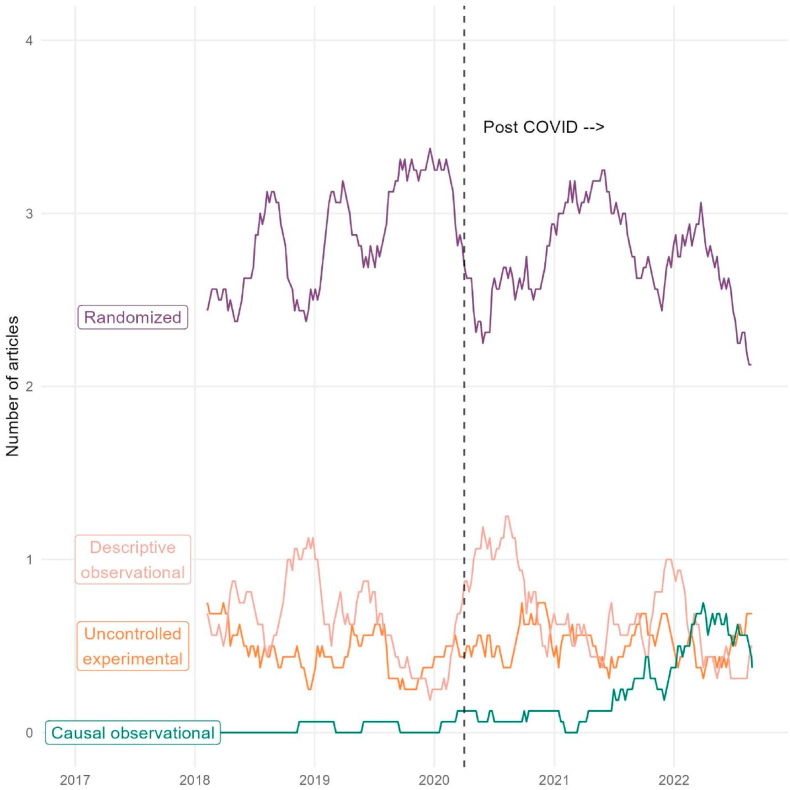
16-week (4 month) moving-average number of original article publications in The New England Journal of Medicine over time by study type.

Within our analysis time period, the 16-week rolling average of the number of weekly articles reached a maximum of 3.38 on 12/19/2019 for randomized trials (Fig. 2). Uncontrolled experimental studies reached a maximum of 0.75 publication on multiple occasions.

Descriptive observational studies reached a peak of 1.25 publication twice in August 2020, and observational studies making a causal conclusion reached a peak of 0.75 publications on March 31, 2022.

Median Altmetric score by study type differed significantly (p-value: <0.001). Observational studies making a causal conclusion had the highest score of 1970 (IQR 931–5591), followed by descriptive observational studies (463, IQR: 172–1106), uncontrolled experimental studies (452, IQR: 233–797), and randomized trials (395 IQR: 242–725). Significance was detected between observational studies making a causal conclusion and randomized trials (p-value <0.001).

**Fig. 3:**
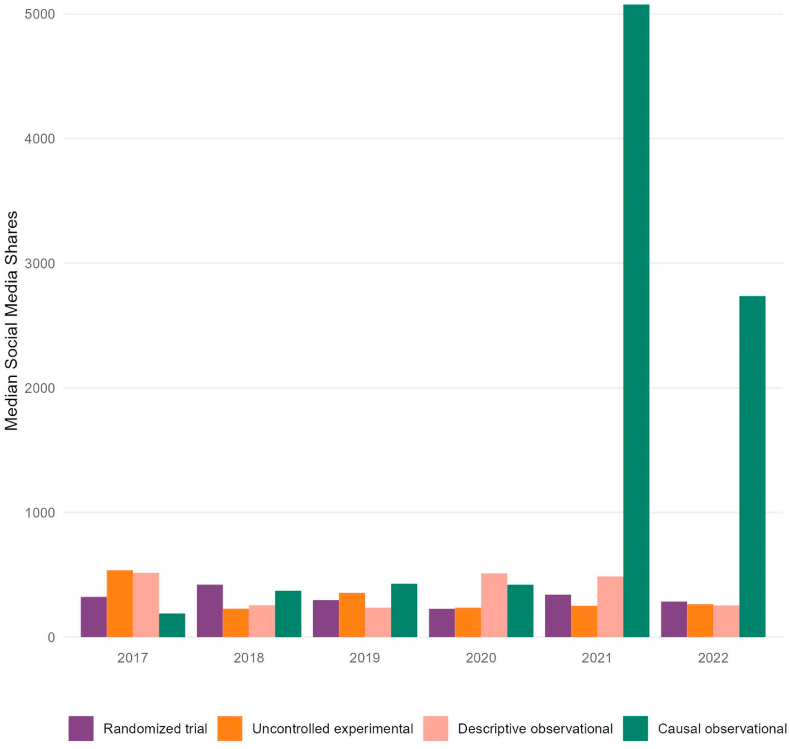
Median number (interquartile range) of social media shares per original article published in The New England Journal of Medicine, by year* and study type *yearly median shares for 2017 & 2022 only represent articles in analysis and do not encompass all 52-weeks within the year.

Stratified by year and methodology, observational studies making a causal conclusion in 2021 had the highest median number of social media shares (Fig. 3, 5076 shares, n = 13, IQR: 1832–11252). Causal observational in 2022 (2736 shares, IQR: 1164–4785, n = 19 articles) and uncontrolled experimental in 2017 (537 shares, IQR: 433–970, n = 9 articles) studies had the next highest, respectively.

Similarly, observational studies making a causal conclusion in 2021 had a median of 3.19 weekly-average citations (Supplement Fig. S1, IQR: 2.17–7.03, n = 13), the highest among all years and methodology types. The one causal observational study of 2018 had the second highest with 1.85 weekly-average cites. Descriptive observational studies in 2021 had 1.73 (IQR: 0.35–5.54) median weekly average cites and the third highest overall.

Randomized trials had a median of 21 authors per article (IQR: 14–30). Uncontrolled experimental studies had a median of 22 authors per article (IQR: 16–32, p-value = 0.40). Descriptive observational studies had a median of 18 authors per article (IQR: 11–31, p-value = 0.05), and observational studies making a causal conclusion had a median of 12 authors per article (IQR: 9–26, p-value = 0.01).

All articles categorized as observations studies making a causal claim are listed in Table 2.

**Table 2:** Observational studies making a causal conclusion published as original article in The New England Journal of Medicine between October 26, 2017 and August 27, 2022.

Article	Date
Mechanical or Biologic Prostheses for Aortic-Valve and Mitral-Valve Replacement	November 9, 2017
Survival after Minimally Invasive Radical Hysterectomy for Early-Stage Cervical Cancer	October 31, 2018
Five-Year Outcomes of Gastric Bypass in Adolescents as Compared with Adults	May 16, 2019
Vaccination of Infants with Meningococcal Group B Vaccine (4CMenB) in England	January 22, 2020
Association of Aspirin with Hepatocellular Carcinoma and Liver-Related Mortality	March 11, 2020
Observational Study of Hydroxychloroquine in Hospitalized Patients with Covid-19	May 7, 2020
HPV Vaccination and the Risk of Invasive Cervical Cancer	September 30, 2020
Life Expectancy after Bariatric Surgery in the Swedish Obese Subjects Study	October 14, 2020
Convalescent Plasma Antibody Levels and the Risk of Death from Covid-19	January 13, 2021
BNT162b2 mRNA Covid-19 Vaccine in a Nationwide Mass Vaccination Setting	February 24, 2021
Multisystem Inflammatory Syndrome in Children — Initial Therapy and Outcomes	June 16, 2021
Treatment of Multisystem Inflammatory Syndrome in Children	June 16, 2021
Prevention and Attenuation of Covid-19 with the BNT162b2 and mRNA-1273 Vaccines	June 30, 2021
Effectiveness of Covid-19 Vaccines against the B.1.617.2 (Delta) Variant	December 8, 2021
Effectiveness of an Inactivated SARS-CoV-2 Vaccine in Chile	February 9, 2021
Protection of BNT162b2 Vaccine Booster against Covid-19 in Israel	July 10, 2021
Effectiveness of Covid-19 Vaccines in Ambulatory and Inpatient Care Settings	July 10, 2021
Waning of BNT162b2 Vaccine Protection against SARS-CoV-2 Infection in Qatar	September 12, 2021
Effectiveness of mRNA Covid-19 Vaccine among U.S. Health Care Personnel	September 22, 2021
BNT162b2 Vaccine Booster and Mortality Due to Covid-19	December 8, 2021
Protection against Covid-19 by BNT162b2 Booster across Age Groups	December 8, 2021
Comparative Effectiveness of BNT162b2 and mRNA-1273 Vaccines in U.S. Veterans	December 1, 2021
Covid-19 Vaccine Effectiveness in New York State	December 1, 2021
Duration of Protection against Mild and Severe Disease by Covid-19 Vaccines	January 12, 2022
Mosquito Net Use in Early Childhood and Survival to Adulthood in Tanzania	March 2, 2022
Effectiveness of BNT162b2 Vaccine against Critical Covid-19 in Adolescents	February 2, 2022
Effect of Covid-19 Vaccination on Transmission of Alpha and Delta Variants	January 5, 2022
Effectiveness of Covid-19 Vaccines over a 9-Month Period in North Carolina	January 12, 2022
Protection against SARS-CoV-2 after Covid-19 Vaccination and Previous Infection	February 16, 2022
Effectiveness of the BNT162b2 Vaccine after Recovery from Covid-19	February 16, 2022
Covid-19 Vaccine Effectiveness against the Omicron (B.1.1.529) Variant	March 2, 2022
Fourth Dose of BNT162b2 mRNA Covid-19 Vaccine in a Nationwide Setting	April 13, 2022
Protection by a Fourth Dose of BNT162b2 against Omicron in Israel	April 5, 2022
Effect of mRNA Vaccine Boosters against SARS-CoV-2 Omicron Infection in Qatar	March 9, 2022
BNT162b2 Protection against the Omicron Variant in Children and Adolescents	March 30, 2022
Protection and Waning of Natural and Hybrid Immunity to SARS-CoV-2	May 25, 2022
Effects of Previous Infection and Vaccination on Symptomatic Omicron Infections	June 15, 2022
Maternal Vaccination and Risk of Hospitalization for Covid-19 among Infants	June 22, 2022
BNT162b2 Vaccine Effectiveness against Omicron in Children 5–11 Years of Age	June 29, 2022
Effectiveness of BNT162b2 Vaccine against Omicron in Children 5–11 Years of Age	July 20, 2022

### 3.1 COVID-19 original articles

Studies concerning COVID-19 made-up 11 % (n = 117) of all publications. COVID-19 studies constituted 7.7 % (n = 55) of all randomized trials, 3.1 % (n = 4) of uncontrolled experimental studies, 17 % (n = 29) of descriptive observational studies, and 72 % (n = 29) of observational studies making a causal conclusion. Differences were significant (p-value <0.001).

**Fig. 4:**
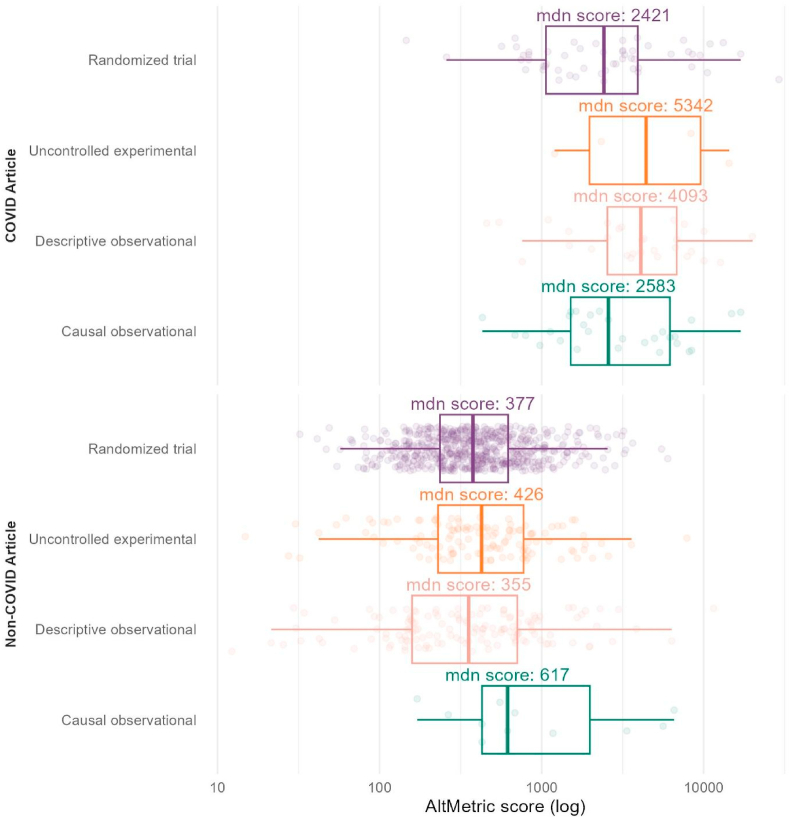
Median number of social media shares for original articles published in The New England Journal of Medicine, per study type and COVID-19 topic.

For COVID-19 articles, median weekly citation was 5.68 (Table 3, IQR: 2.50–9.21, p-value <0.001), and the median Altmetric score was 2999 (IQR:1387–6297, p-value <0.001). Randomized COVID-19 trials had a median Altmetric score of 2421 (Fig. 4, IQR: 1063–3920), uncontrolled experimental studies had a median score of 5432 (IQR: 2049–9851, p-value = 0.36), descriptive observational studies had a median score of 4093 (IQR: 2545–6823, p-value = 0.04), and observational studies making a causal conclusion had a median score of 2583 (IQR: 1513–6197, p-value = 0.42).

**Table 3:** Characteristics of original articles published in The New England Journal of Medicine between October 26, 2017 and August 27, 2022, stratified by COVID-19 vs non-COVID-19 topic.

	COVID-19 article, N = 117	non-COVID-19 article, N = 934	p-value^a^
Weeks since April 1, 2020, median (IQR)	71 (37–93)	−12 (-69–55)	<0.001
Time period, before/after COVID, n (%)			<0.001
Post	114 (97)	422 (45)	
Pre	3 (2.6)	512 (55)	
Methodology, n (%)			<0.001
Randomized trial	55 (47)	660 (71)	
Uncontrolled experimental	4 (3.4)	124 (13)	
Descriptive observational	29 (25)	139 (15)	
Causal observational	29 (25)	11 (1.2)	
Weekly citations, median (IQR)	5.68 (2.50–9.21)	1.03 (0.50–2.04)	<0.001
Altmetric score, median (IQR)	2999 (1387–6297)	380 (223–661)	<0.001
Social media shares, median (IQR)	2890 (1151–7716)	270 (155–504)	<0.001
Brief reports, n (%)	4 (3.4)	45 (4.8)	0.7
# of authors, n (%)	26 (14–37)	21 (13–30)	0.012

a Wilcoxon rank sum test; Pearson's Chi-squared test.

Randomized COVID-19 trials had a median of 5.96 (Supplement Fig. S2, IQR: 3.14–8.96) weekly-average citations, and randomized non-COVID-19 trials had a median of 1.11(IQR: 0.57–2.18). Median weekly citations per causal COVID-19 observational study was 2.5 (IQR: 1.56–3.65), compared to 1.3 (IQR: 0.91–1.87) for non-COVID-19 observational studies making a causal conclusion. Differences in median weekly citations between COVID-19 and non-COVID-19 articles were significant for both randomized trials (p-value <0.001) and causal studies (p-value = 0.02). Differences in median weekly citations between randomized and observational studies making a causal conclusion were only significant among COVID-19 articles (p-value = 0.01).

## 4 Discussion

We examined approximately 5 years of original articles in NEJM, before and during the COVID-19 pandemic.

First, prior to COVID19, observational studies making a causal claim were seldom published in NEJM. With just 5 such articles constituting 1 % of NEJM original publications pre-COVID-19. After the beginning of the pandemic, these studies made-up 6.5 % (n = 35) in the post COVID-19 era. Unlike other classes of articles, observational studies making a causal conclusion mostly concerned COVID-19 (72 % vs. 11 % overall). Naturally, there are many scientific questions introduced by a pandemic that require causal inference but may not be amenable to randomization, yet the fidelity of this method remains unknown, as we describe below.

Historically, observational studies have shown poor concordance with randomized literature. In a 2001 paper, researchers found that the conclusion of the two methods differed in 15 % of occurrences where both study designs had been performed [6]. Others have asked if the use of propensity score matching would improve concordance. However, a comparison of propensity score weighted and matched studies to RCTs failed to validate this hypothesis [12]. More recently, Kumar and colleagues performed propensity score weighted analyses to generate 141 observational studies for which randomized trials already existed [7]. This project found a poor replication rate. Just 45 % percent reached the same therapeutic conclusion [8].

Proponents of observational research to yield causal conclusions have generated novel methods to improve their reliability. Hernán and colleagues pioneered the use of the target trial framework which has yielded important results on the topics of statins and hormone therapy [11,13,14]. Recently the US Food and Drug Administration commissioned project “RCT Duplicate” to test the target trial framework. Unfortunately, RCT Duplicate has found poor concordance between the two methods [15]. For 10 RCTs, trial emulation could only replicate the regulatory conclusion in 6 cases (60 %) – providing a little better than chance agreement. As such, our concern with the rise of observational studies making a causal conclusion in NEJM is not whether answers to these questions are needed – they are – but whether they are reliable.

Our analysis expands upon previous research that found early, high-impact literature for COVID-19 was primarily case-series, a methodology generally considered inferior to those utilized by its non-COVID-19 counterparts [16,17]. Although lower-tier evidence early in the pandemic was surely inevitable, given the novelty of the situation and the limited understanding of the virus, we find that as time progressed, high-quality data, based on randomized data, did not appear to significantly replace such studies, and, contrarily, reliance on the method grew.

Our second finding is that the impact of observational studies making causal conclusions is non-negligible. COVID-19 has led to an explosion of interest in the scientific literature. Impact of COVID-19 articles outweighed non-COVID-19 papers, both through social media and citations (median social shares: 2890 vs. 270, median weekly citations: 5.68 vs. 1.03, for COVID-19 and non-COVID-19 respectively). However, the Altmetric scores of COVID-19 articles found that the impact of randomized COVID-19 trials and causal observational COVID-19 studies were not significantly different.

Randomized COVID-19 trials had a significantly higher median of weekly-average citations than causal observational COVID-19 studies. This pattern was not seen in the non-COVID-19 studies, as median weekly citations were similar across methodology types. Overall, COVID-19 observational studies (causal and descriptive) had higher median weekly-average citations than non-COVID-19 studies of all methodologies, suggesting COVID-19 as a greater driving factor in the rise rather than methodology. However, the rise highlights a changing trend of greater dissemination for observational studies making a causal conclusion that has yet to fully deflate to the prior baseline. This is true for both informal discourse (as seen in social media metrics) and knowledge building (seen in the average weekly citations).

There is little literature to suggest the specific mechanisms behind these trends. Previous research found that a shortened review time for COVID-19-article may have resulted in laxities in the peer-review process, skyrocketing the number of studies listed in PubMed [2,18]. However this does not explicitly explain the change in the types of methodology over time. Our findings show slightly fewer authors per article among observational studies making a causal conclusion in comparison to randomized trials. Overall, the increased size of research teams, the publish-or-perish dogma of academia, and the profit driven incentives of scholarship, are all longstanding critiques of the academic publishing system and we speculate that these aspects may have worsened under the strain of the pandemic [[19], [20], [21], [22]]. However preliminary research is needed to explore these factors in their relation to the COVID-19 pandemic response.

## 5 Limitations

Our study has at least 3 limitations. First, we chose NEJM because it is the highest impact factor medical journal and has shaped pandemic thinking, but it is likely not representative of broader scientific research. As such, we would not extrapolate our findings beyond this journal. Nevertheless, the findings have importance given high Altmetric scores. Second, we categorized studies broadly by methodology but did not conduct a thorough assessment of study quality. It is possible that some randomized trials are inferior to other observational studies making a causal conclusion. However, to our knowledge no group has provided a set of benchmarks that would permit investigators to sort this out, and furthermore, empirical comparisons between the two still show marked disagreement. As such, our paper broadly aligns with data from prior publications that have reported on the levels of evidence for research methodology [23]. Third, our classification of study type could have subjectivity, and as such, all articles were blindly reviewed by two independent reviewers (JT and AH), and the final list of included observational studies making a causal conclusion was verified by a third person (VP). It is possible others may classify articles differently, and we encourage other research teams to replicate our efforts and expand upon them.

## 6 Conclusion

Prior to the start of the COVID19 pandemic, observational literature making specific causal conclusions or inferences regarding medical products or strategies was seldom published in the *NEJM*, but since the start of the pandemic, it now comprises more than 1 in 20 original articles. This research has had massive reach, through both social media and subsequent citations. Whether these papers represent true causal estimates remains uncertain. As COVID-19 was the first emergency pandemic for the United States general public within the modern age, further understanding of science dissemination is critical in combating future public health emergencies. To assist in the dissemination of more correct information, editors and reviewers should monitor and encourage language in published manuscripts that is appropriately supported by the methodology used to derive results and conclusions.

## Funding

This research is not funded.

## Data availability

The datasets used and/or analyzed during the current study are available from the corresponding author on reasonable request.

## CRediT authorship contribution statement

**Alyson Haslam:** Writing – review & editing, Writing – original draft, Investigation, Formal analysis, Data curation. **Vinay Prasad:** Writing – review & editing, Writing – original draft, Investigation, Formal analysis, Data curation, Conceptualization.

## Declaration of competing interest

The authors declare the following financial interests/personal relationships which may be considered as potential competing interests:

Dr. Prasad receives research funding from 10.13039/100014848Arnold Ventures through a grant made to 10.13039/100008069UCSF, and royalties for books and writing from 10.13039/100007880Johns Hopkins Press, MedPage, and the Free Press. He declares consultancy roles with UnitedHealthcare and OptumRX; He hosts the podcasts, Plenary Session, VPZD, Sensible Medicine, writes the newsletters, Sensible Medicine, the Drug Development Letter and VP's Observations and Thoughts, and runs the YouTube channel Vinay Prasad MD MPH, which collectively earn revenue on the platforms: Patreon, YouTube and Substack.
