# Supplementary material for: Publication of observational studies making claims of causation over time
Source: Contemp Clin Trials Commun. 2024 Jun 17;40:101327. doi: 10.1016/j.conctc.2024.101327 (PMC11259777; doi:10.1016/j.conctc.2024.101327)

**SUPPLEMENT**

**Figure S1: Median (interquartile range) weekly average citations of social media shares for original articles published in The New England Journal of Medicine, by year* and study type**


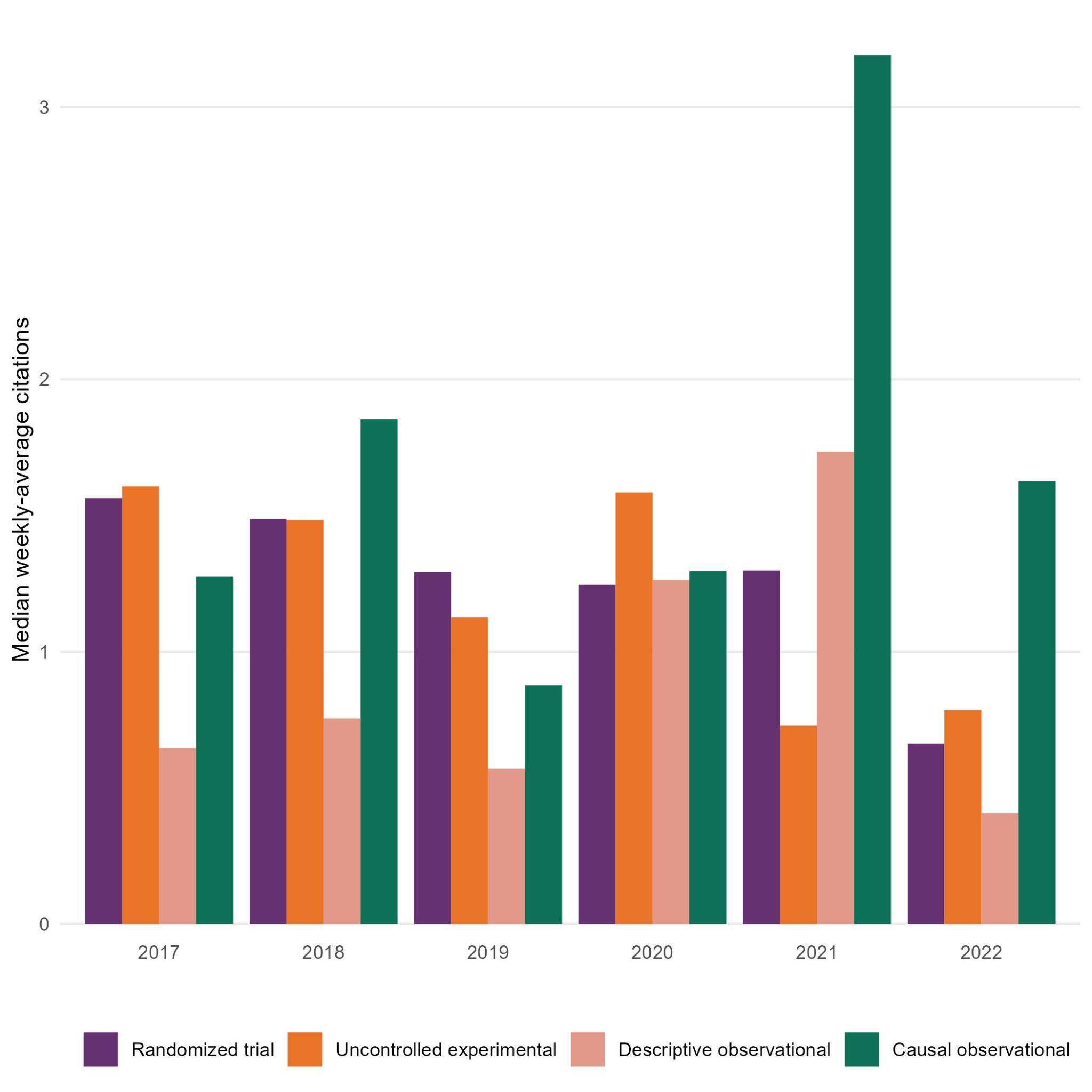


*yearly median shares for 2017 & 2022 only represent articles in analysis and do not encompass all 52-weeks within the year

**Figure S2: Median, weekly-average citations for original articles published in The New England Journal of Medicine, by COVID**-19 **topic and study type category***

*Figure excludes n = 8 articles higher than 30 weekly citations.


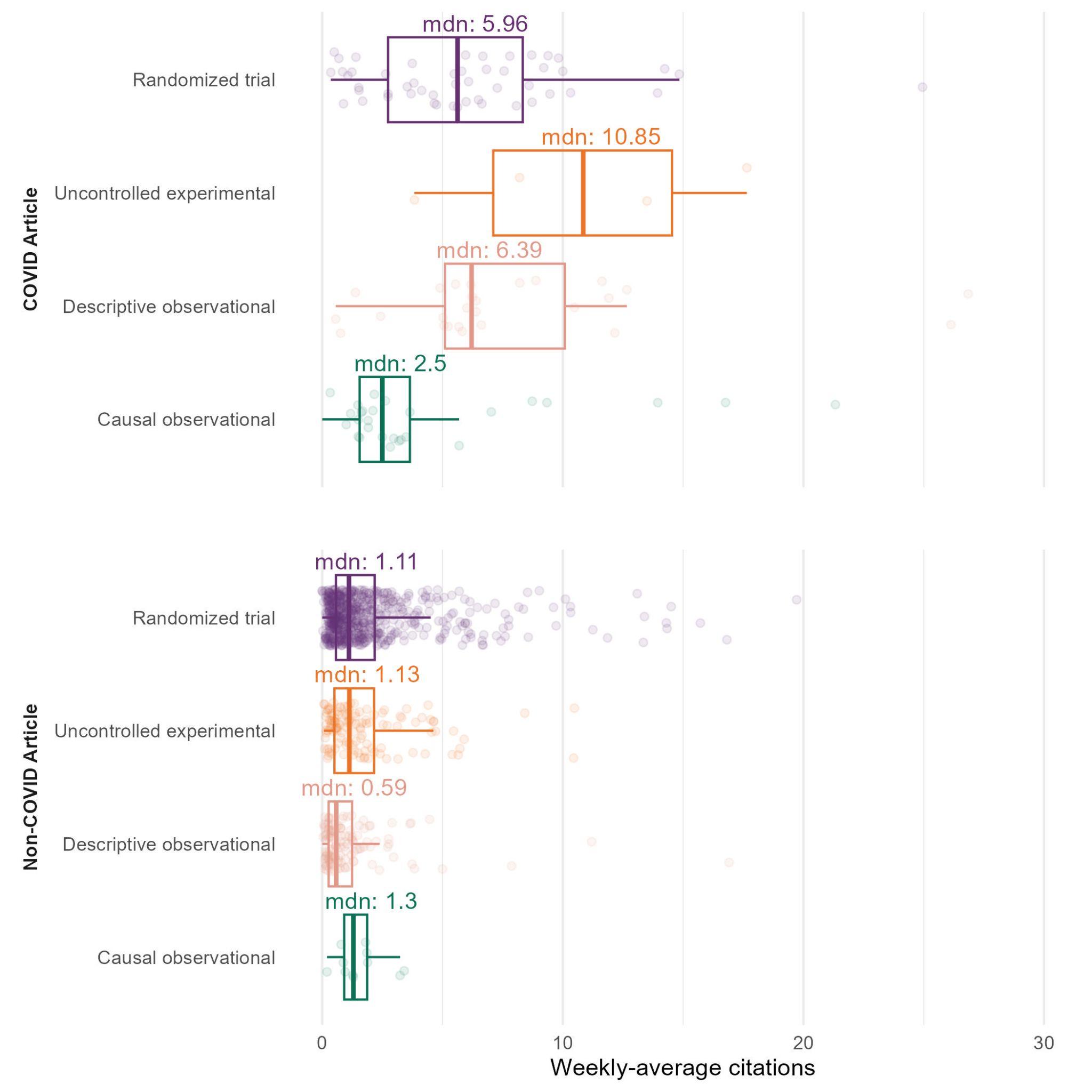

Supplement: Multimedia component 1 [file mmc1.docx]
